# Updates on Management of Avascular Necrosis Using Hip Arthroscopy for Core Decompression

**DOI:** 10.3389/fsurg.2022.662722

**Published:** 2022-04-08

**Authors:** Kyle N. Kunze, Spencer W. Sullivan, Benedict U. Nwachukwu

**Affiliations:** Sports Medicine Institute, Hospital for Special Surgery, New York, NY, United States

**Keywords:** avascular necrosis, osteonecrosis, hip preservation, core decompression, outcomes

## Abstract

Osteonecrosis of the femoral head (ONFH) is caused when circulation within the femoral head is disrupted. Etiology of this disease is characterized by either traumatic events or atraumatic risk factors, such as chronic alcohol consumption or glucocorticoid use. ONFH commonly presents as pain in the groin, gluteus muscles, and/or knee with reductions in internal rotation range of motion of the hip. ONFH pathology can be confirmed with radiographic or advanced imaging and characterized by stage, size and location of the lesion. Treatment for ONFH consists of conservative and therapeutic methods as well as surgical intervention. Historically, ONFH has been treated using total hip arthroplasty (THA), but with increasing incidence in a younger, active population, measures to preserve the native hip joint have been explored. Recent advancements in hip arthroscopy and arthroscopy-assisted core decompression have led to improved outcomes, reduced pain and higher hip survival rate for early onset ONFH compared with more invasive approaches such as THA. Biologic treatments combined with arthroscopic core decompression have also shown improved outcomes and quality of life in few reports, suggesting a potential role for biologic adjuncts. The current study provides a comprehensive review and update on the literature surrounding arthroscopy-assisted core decompression for patients with ONFH.

## Introduction/Background

Osteonecrosis of the femoral head (ONFH) is characterized by apoptosis of osteoblasts and osteoclasts as well as mesenchymal and hematopoietic stem cells (responsible for generation of bone marrow) within the femoral head and neck, also deemed avascular necrosis (AVN). AVN is commonly caused by the interruption of blood supply to these specialized bone cells (1, 2). Without proper vascularization, loss of cancellous bone and articular cartilage leads to changes in the shape of the femoral head in addition to underlying subchondral deformities (2, 3). Thus, the physiologic changes and loss of bone have anatomic consequences on the structure of the femoral head and acetabular junction, and this may result in increasing pain and dysfunction.

ONFH is designated by two major categories: atraumatic and traumatic. Atraumatic ONFH is highly associated with alcohol and glucocorticoid use and is influenced by a number of other genetic, metabolic and physical blood flow restrictive factors (4, 5). Traumatic cases of ONFH often result from clear etiology, causing physical restriction and/or occlusion of blood supply to the femoral neck and head (4, 6). ONFH is diagnosed most commonly in active individuals between the age of 20–40 years (7, 8) and, if left untreated in the early stages, may progress to result in total hip arthroplasty (THA). An estimated 5–12% of THA procedures performed in the United States have been attributed to untreated ONFH (4, 7). Although THA has historically been successfully used to treat ONFH, core decompression and other hip preservation techniques have been shown to improve outcomes and quality of life postoperatively while allowing for maintenance of the native hip joint, reducing lifetime risk of revision (9, 10).

Despite increasing literature pertaining to the use of arthroscopic-assisted core decompression and decompression in conjunction with arthroscopic hip preservation surgery, a comprehensive evaluation of the literature is lacking. As these procedures continue to be performed, it will be imperative to assess the outcomes associated with these procedures as to critically evaluate and reassess the justification for their use. The purpose of the current study was to provide a comprehensive review and update on the literature surrounding arthroscopy-assisted core decompression for patients with ONFH.

## Indications for Surgery

It is critical to perform a comprehensive history and physical examination in order to diagnose ONFH; in particular, questions should be directed at risk factors for OFNH in patients that present with hip pain, including history of alcohol and chronic steroid use, recent travel, medical and family history of genetic disease or Sickle cell anemia, history of trauma or irradiation and surgical history including procedures which may have jeopardized blood flow to the femoral head and neck. Pain is often reported in the groin, glute and/or knee with exacerbated pain in internal rotation of the hip (11).

On physical examination, considerable loss of internal range of motion may indicate late stage ONFH and collapse of the femoral head (12). However, range of motion restrictions will often be non-specific and patients may also demonstrate examinations consistent with femoroacetabular impingement (FAI), iliopsoas tendinitis or osteoarthritis of the hip. The use of radiographic and advanced imagining is imperative to help discern the primary etiology of the hip and groin pain. The severity of ONFH has been described through a number of classification systems supported through radiographic evidence and MRI (13). Anteroposterior (AP) and cross-table or Dunn lateral radiographs will demonstrate sclerosis, subchondral cysts or a crescent sign in early stages of ONFH, whereas more severe stages will demonstrate collapse or flattening of the femoral head, joint space narrowing and eventually severe degeneration. MRI may reveal subchondral edema or the double-density sign consistent with areas of revascularization and bone formation.

Several systematic reviews and meta-analyses have found the Ficat classification scale is most widely used for grading severity of ONFH followed by the University of Pennsylvania/modified Ficat (Steinberg) systems (14, 15). In the early stages of OFNH, radiographs and advanced imaging may be unrevealing, such as in Steinberg grade 0-I scenarios (12). However, early ONFH can be detected with high sensitivity (93%) and specificity (91%) using magnetic resonance imaging (MRI) (16). Arthroscopic-assisted core decompression or core decompression in conjunction with arthroscopic hip preservation surgery is indicated in pre-collapse stages including Ficat grades 0-II and Steinberg grades 0-III OFNH severity with or without soft-tissue or osseous pathology, such as labral tears and FAI. We do not recommend arthroscopic treatment of more severe OFNH stages as arthroscopic-assisted decompression may fail to confer meaningful improvements in bone quality and function and has a high rate of conversion to THA.

## Outcomes

Arthroscopic-assisted core decompression of the femoral head involves creating standard hip arthroscopy portals and performing a diagnostic arthroscopy. This allows the surgeon to identify other common intra-articular pathologies at the time of decompression, such cam-deformities, labral defects, and chondral defects (17). Through an additional distal and posterior portal, the femoral head can be accessed with guide wires allowing for subsequent reaming and intra-osseous debridement. Following replacement of the intra-osseous cavity with a synthetic bone graft substitutes and/or biologic adjuncts such as bone marrow aspirate, the articular side of the femoral head can be explored to confirm absence of penetration into the hip joint. Despite several established arthroscopic techniques for core decompression with or without treatment of additional intra-articular pathology, large series on patient outcomes remain scarce (18–23).

Theopold et al. (24) reported the outcomes of seven patients with ONFH treated with arthroscopically navigated core decompression of the femoral head using an established optoelectronic system with fluoro-free software module. They noted that over 50% of patients had an additional labrum lesion which they were able to repair at the time of surgery, suggesting that a major benefit of arthroscopically assisted core decompression is the ability to identify and treat additional pathologies. Similarly, Nazal et al. (25) reported on the minimum 5-year outcomes of arthroscopic-assisted treatment of patients with pre-collapse ONFH (Ficat grade 0-II). A total of 11 hips were included, of which 73% necessitated labral repair or debridement and 64% necessitated microfracture at the time of surgery. At a mean 7-year follow-up, the majority (54.5%) of hips had not converted to THA, and there were no major complications, such as sub-trochanteric fracture or violation of the articular cartilage, as well as no minor complications as a result of the procedures. However, this study also suggests that arthroscopic core decompression is most reliable in patients in early stages of ONFH. According to Ficat-Arlet staging of the hip, 100% of hips preoperatively typed as Stage IIb converted to THA within the 7 year mean follow-up period as opposed to 25% in the Stage IIa cohort and 0% in the Stage I cohort, respectively (25). Furthermore, both studies did not report follow-up in the form of patient reported outcomes. Future research should determine the patient subjective experience following arthroscopic-assisted core decompression for early-onset ONFH.

Guo et al. (21) performed a randomized controlled trial where patients with Ficat grade II ONFH were treated with arthroscopic-guided core decompression and bone grafting combined with selective arterial infusion (experimental group, *n* = 35) or percutaneous core decompression combined with selective arterial infusion (control group, *n* = 41). At a mean 30-month follow-up, the mean Harris Hip Score (HHS) was significantly greater in the experimental group compared with the control group (86.7 vs. 78.6, *p* < 0.05), despite similar reported HHS preoperatively. They also reported that the change in the radiographic appearance of the femoral head was significantly better in the experimental group. Although both treatment methods are effective, the authors concluded that arthroscopic-guided core decompression can obtain better results as the necrotic femoral head can be positioned and scraped more accurately.

Ellenrieder et al. (19) reported the outcomes of 53 patients (56 hips) with Steinberg grade 0-IVa ONFH treated with arthroscopically-assisted core decompression. At a mean 33-month follow-up, the success rate (defined as no conversion to THA, no reoperations, or no radiological progression of OFNH with clinical symptoms) was 86%. Of the nine failures, the majority were in stage IVa (31%) and stage III (25%) patients. These results support the indication of utilizing arthroscopic-assisted core decompression in late-stage ONFH with increased risk of failure and/or progression to THA. Additionally, we would like to introduce the case of a 46-year-old female who presented to our service with early subchondral collapse and subsequently underwent core decompression and labral debridement, the need for the latter of which was identified during the diagnostic arthroscopy. The patient remains free of major complication at the 2-year follow-up point as defined by any revision procedures or progression to THA. Such cases bolster the need for further research on the efficacy of core decompression in patients with more advanced AVN.

Guadilla et al. (20) studied the use of arthroscopic-assisted core decompression and platelet-rich plasma (PRP) therapy in four patients with pre-collapse ONFH. At a mean 14-month follow-up, all patients reported a reduction in pain intensity and returned to their “normal style of life” by 5 months. Two patients were found to have labral tears and were treated with debridement. The authors noted that this procedure may improve the overall diagnostic accuracy of labrum degeneration or other pathologies in patients with ONFH when not identified on MRI and also allows for precise decompression due to enhanced visual control.

The above literature suggests that arthroscopic-assisted core decompression is a promising and efficacious treatment option in the treatment of pre-collapse stages of ONFH with or without additional intra-articular pathology. However, based on the paucity of literature and identification of only one randomized trial, there is currently only weak evidence to support its use and additional, high-quality trials are needed. In this current review, only one study had reported patient reported outcomes, the HHS at a mean 30-month follow-up. Future studies should incorporate patient reported outcomes at regular postoperative follow-up to determine subjective patient experience following arthroscopic-assisted core decompression.

## Conclusion

Arthroscopic-assisted core decompression for OFNH has several purported benefits including the ability to visual the joint surface to avoid penetration, perform diagnostic arthroscopy and address concomitant intra-articular pathology, and the potential for more comprehensive and accurate debridement of the avascular bone lesions. Based on the available literature, this procedure may effectively reduce pain and increase survivorship of the native hip in patients with pre-collapse ONFH prior, with better outcomes in earlier pre-collapse stages. There is inadequate evidence to recommend the use of biologic adjuncts such as platelet-rich plasma in conjunction with these procedures. Future research is imperative to continue to understand the indications and outcomes of arthroscopic-assisted core decompression.

## Author Contributions

SS was the main author of this mini-review, performing the literature search and drafting/revising of this article. KK played a significant role in the revisions and writing of this manuscript as well as literature search for the final product. BN formulated and designed the outline of this mini-review and provided crucial editing and supervision to the final manuscript. All authors contributed to the article and approved the submitted version.

## Conflict of Interest

BN declares ownership interest in BICMD (founder) outside of the scope of this manuscript. The remaining authors declare that the research was conducted in the absence of any commercial or financial relationships that could be construed as a potential conflict of interest.

## Publisher's Note

All claims expressed in this article are solely those of the authors and do not necessarily represent those of their affiliated organizations, or those of the publisher, the editors and the reviewers. Any product that may be evaluated in this article, or claim that may be made by its manufacturer, is not guaranteed or endorsed by the publisher.
